# The United Kingdom Childhood Cancer Study

**DOI:** 10.1054/bjoc.1999.1031

**Published:** 2000-02-01

**Authors:** L J Kinlen


*British Journal of Cancer* (2000) **82**(5), 999
© 2000 Cancer Research Campaign
DOI: 10.1054/ bjoc.1999.1031, available online at http://www.idealibrary.com on IDEAL®

Editorial

### The United Kingdom Childhood Cancer Study

**L.J. Kinlen**

The article in this issue on the recently completed national study of the aetiology of cancer in children is dedicated to the memory of Professor Gerald Edward Adams, editor-in-chief of the Journal from 1994 until his untimely death on 6 June two years ago. Ged, as he was universally known to his colleagues and friends, was one of the small group who, in 1990, had had the idea for a study of children with cancer that would both include a large enough number of cases and be extensive enough in the topics it covered to be able to test the relevance of the many suggestions that had been made about the possible causes of the disease. But he was responsible for much more than that. For he undertook, in his capacity as Chairman of the Radiation and Cancer Sub-Committee of the UK Coordinating Committee on Cancer Research, to raise the funds that would enable the study to be carried out and he served on the committee that planned the study in detail, organized its conduct on a regional basis, and oversaw its progress. His enthusiasm inspired all who worked on the study and knitted together a group of independent-minded scientists from different disciplines into an amicable and effective team. Sadly he never saw any of the results of the work which began to be analysed only a few months after his death.

The article on the UK Childhood Cancer Study describes its origins and purpose, the patients studied, the data obtained, and the methods by which the information has been secured and we hope to publish reports on its findings on many specific aspects in due course. It is unusually long for this Journal, reflecting both the complexity of the study and the importance we attach to it. The coverage of 11 400 families, more than 3800 of them involving a case of childhood cancer, represents a massive undertaking in which literally hundreds of people collaborated. Few national case–control studies have been carried out of any disease in Britain and fewer still of childhood cancer of comparable size anywhere. The pioneering Oxford Survey of Childhood Cancer, started in 1953 (Stewart et al, 1958), was larger, but it did not collect blood samples, nor could it, obviously, cover the many factors that have become of potential interest only in more recent years. More cases of acute lymphoblastic leukaemia were included in a recent US study (Linet et al, 1997) which was limited to leukaemia and did not cover other childhood malignancies. The UK study is exceptional by any standards, not only in its size and national scope, but also in covering so many factors, including exposure to electro-magnetic fields and radon, and the collection of biological material. For the last will provide information on HLA types for all malignancies and permit the leukaemias to be classified by immunophenotype, cytogenetics and abnormalities of DNA.

### REFERENCES

Linet MS, Hatch EE, Kleinerman RA, Robison LL, Kaune WT, Friedman DR, Severson RK, Haines CM, Hartstock CT, Niwa S, Wacholder S and Turone RE (1997) Residential exposure to magnetic fields and acute lymphoblastic leukaemia in children. *N Engl J Med* **337**: 1–7

Stewart A, Webb J and Hewitt D (1958) A survey of childhood malignancies. *Br Med J* 1495–1508

